# Bacterial cell factories for recombinant protein production; expanding the catalogue

**DOI:** 10.1186/1475-2859-12-113

**Published:** 2013-11-18

**Authors:** Neus Ferrer-Miralles, Antonio Villaverde

**Affiliations:** 1 Institut de Biotecnologia i de Biomedicina, Universitat Autònoma de Barcelona, Bellaterra 08193 Barcelona, Spain; 2 CIBER de Bioingeniería, Biomateriales y Nanomedicina (CIBER-BBN), Bellaterra 08193 Barcelona, Spain; 3 Departament de Genètica i de Microbiologia, Universitat Autònoma de Barcelona, Bellaterra 08193 Barcelona, Spain

## 

*Escherichia coli* has been the pioneering host for recombinant protein production, since the original recombinant DNA procedures were developed using its genetic material and infecting bacteriophages. As a consequence, and because of the accumulated know-how on *E. coli* genetics and physiology and the increasing number of tools for genetic engineering adapted to this bacterium, *E. coli* is the preferred host when attempting the production of a new protein. Also, it is still the first choice for protein production at laboratory and industrial scales for an important number of proteins, being fast growth and simple culture procedures critical issues. When searching for an ideal system for protein production, this bacterial species is clearly far from offering, in generic terms, optimal conditions for protein production and downstream. Plasmid loss and antibiotic-based maintenance, undesired chemical inducers of gene expression, plasmid/protein-mediated metabolic burden and stress responses, lack of post-translational modifications (including the inability to form disulphide bonds), none or poor secretion, protein aggregation and proteolytic digestion, endotoxin contamination and complex downstream are among the main obstacles encountered during protein production in *E. coli*. In the pharmaceutical scenario, proper protein glycosylation is often requested and simplest purification procedures become highly desirable when pursuing cost-effective bioproduction. In this context, the yeast *Sacharomyces cerevisae*, diverse mammalian cell lines, insect cells and whole plant and animals (as transgenic systems) are being incorporated to the protein production scenario [1], and many of these products have been already approved for use as protein drugs [2]. Other (less conventional) yeast species and a more limited number of species of filamentous fungi [3], molds [4], moss [5], algae [6] and protozoa [7] are also under development as potential suppliers of recombinant proteins. The engineering of such systems could represent a promising way to the cost effective production of high quality protein versions that biotechnology and biomedical industries are steadily demanding. The potential and versatility of these platforms as protein producers or in general, as cell factories for added value products such as chemicals, amino acids or vitamins has been stressed in recent experimental reports or reviews [8-17]. Despite this, it must be noted that adapting large-scale production processes to the biological complexity of some of these systems might represent, in some cases, an unaffordable task.

From a different angle, bacterial hosts others than *E. coli* are attracting attention as cell factories due to their metabolic diversity and biosynthetic potential derived from adaptation to extremely diverse environments. The most important bacterial groups explored as cell factories for recombinant proteins and their associated potentialities are summarized in Table 1. The implementation of lactic acid bacteria as a routine cell factory expands their applications from conventional food microbiology [18-21] to protein production and also protein drug display and delivery [22-29], taking advantage of the generically recognized as safe (GRAS) features of this platform. Improved solubility in halophillic and cold-adapted bacteria, enhanced secretion in acid lactic bacteria and in general in endotoxin-free gram-positive species and post-translational modifications in mycobacteria among others are highly appealing properties in protein production, that can be of special value for specific difficult-to-express proteins. While exhibiting most of the above mentioned limitations linked to prokaryotic-based production, exploring bacterial species other than *E. coli* should be not abandoned but fully supported as it will not only expand the current catalogue of cell factories but also offer novel process opportunities in easily cultivable/scalable systems that might pose, in generic terms, less methodological issues than unconventional protein production systems [30].

**Table 1:** The most important bacterial groups explored as cell factories for recombinant protein production

	**Host**	**Main features**	**Reviews**^**a**^	**Main bacterial Species**	**Case proteins**	**References**
Proteobacteria	Caulobacteria	Easy purification of secreted RSaA fusions	[31,32]	*Caulobacter crescentus*	Hematopoietic necrosis virus capsid proteins	[33]
					β-1,4-glycanase	[34]
	Phototrophic bacteria	High production of membrane proteins	[35]	*Rodhobacter sphaeroides*	Membrane proteins	[35]
	Cold adapted bacteria	Improved protein folding	[36,37]	*Pseudoalteromonas haloplanktis*	3H6 Fab	[38]
					Human nerve growth factor	[39]
				*Shewanella sp. strain Ac10*	β-Lactamase, peptidases, glucosidase	[40]
	Pseudomonads	Efficient secretion	[41]	*Pseudomonas fluorescens*	Human granulocyte colony-stimulating factor	[42]
				*Pseudomonas putida*	Single chain Fv fragments	[43]
				*Pseudomonas aeruginosa*	Penicillin G acylase	[44]
	Halophilic bacteria	Solubility favored	[45]	*Halomonas elongata*	β-Lactamase	[45]
				*Chromohalobacter salexigens*	Nucleoside diphosphate kinase	[46]
Actinobacteria	Streptomycetes	Efficient secretion	[47,48]	*Streptomyces lividans*	*M. tuberculosis* antigens	[49]
				*Streptomyces griseus*	Trypsin	[50]
	Nocardia	Efficient secretion	[48]	*Nocardia lactamdurans*	Lysine-6-aminotransferase	[51]
	Mycobacteria	Posttranslational modifications	[52]	*Mycobacterium smegmatis*	Hsp65-hIL-2 fusion protein	[53]
					Mycobacterial proteins	[54]
	Coryneform bacteria	High-level production and secretion; GRAS	[48,55]	*Corynebacterium glutamicum*	Protein-glutaminase	[56]
				*Corynebacterium ammoniagenes*	Pro-transglutaminase	[57]
				*Brevibacterium lactofermentum*	Cellulases	[58]
Firmicutes	Bacilli	High-level production and secretion	[59-64]	*Bacillus subtilis*	β-Galactosidase	[65]
				*Bacillus brevis*	Disulfide isomerase	[66,67]
				*Bacillus megaterium*	Antibodies	[68]
				*Bacillus licheniformis*	Subtilisin	[69]
				*Bacillus amyloliquefaciens*	Amylases	[70]
	Lactic acid bacteria	Secretion; GRAS	[22-24,71]	*Lactococcus lactis*	Fibronectin-binding protein A, internalin A, GroEL	[72,73]
				*Lactobacillus plantarum*	β-Galactosidase	[74]
				*Lactobacillus casei*	VP2-VP3 fusion protein of infectious pancreatic necrosis virus	[75]
				*Lactobacillus reuteri*	Pediocin PA-1	[76]
				*Lactobacillus gasseri*	CC chemokines	[77]

^**a**^Generic reviews about the biological platform or about specific tools for protein production.

Towards a progressively more competitive biological synthesis by microbes [78] and assisted by expanding systems metabolic engineering and synthetic biology tools [79], industrial biotechnology should desirably find within the prokaryotic world, a growing spectrum of alternatives to eukaryotic cell factories, that apart from easy and cost-effective cultivation provide unexpectedly high metabolic versatility and biosafety of their protein-based products. In some cases and at a large extent, it is solving some of the main issues posed by *E. coli* as traditional producer or recombinant proteins.

## Competing interests

The authors declare that they have no competing interests.
